# Effect of Adjunctive Low-Level Laser Therapy on Wound Healing Following Surgical Gingival Depigmentation: A Prospective Study

**DOI:** 10.7759/cureus.111748

**Published:** 2026-06-29

**Authors:** Shaik Abdul Riyaz, O Pavan Kumar, Karri Seshu Kumar, Shazia Parveen, K. P. Ashok, Viswanath Mylapoori, Sania Azmi Md, Kadapa Khader Basha

**Affiliations:** 1 Department of Periodontics, Government Dental College and Hospital, Kadapa, IND; 2 Department of Conservative Dentistry and Endodontics, Government Dental College and Hospital, Kadapa, IND; 3 Department of Oral and Maxillofacial Surgery, Government Dental College and Hospital, Kadapa, IND; 4 Department of Oral and Maxillofacial Surgery, Care Dental College, Guntur, IND; 5 Department of Periodontics, KLR's Lenora Institute of Dental Sciences, Rajahmahendravaram, IND; 6 Department of Oral and Maxillofacial Pathology, Care Dental College, Guntur, IND; 7 Department of Dentistry, Shifa Dental Clinic, Mydukur, IND

**Keywords:** gingival pigmentation, gingival surgery, low-level light therapy, pain measurement, wound healing

## Abstract

Introduction

Gingival hyperpigmentation is a common esthetic concern managed by surgical depigmentation. However, delayed epithelialization and postoperative discomfort remain challenges. Low-level laser therapy (LLLT) has been suggested as an adjunct for enhancing healing and reducing pain. This study aimed to evaluate the effect of adjunctive LLLT on wound healing and postoperative pain following surgical gingival depigmentation.

Methods

This prospective observational study included 24 systemically healthy participants with physiological gingival pigmentation. Each participant underwent surgical depigmentation, with selected sites receiving adjunctive LLLT and others serving as the comparison sites. Clinical evaluations were performed on postoperative days 3, 7, and 15. Epithelialization was assessed by the residual wound area (mm²), wound healing was evaluated using a Likert scoring, and pain perception was recorded using a visual analog scale (VAS). Non-parametric tests were applied at a significance level of p < 0.05.

Results

On day 3, the median epithelialization area was significantly lower in the LLLT-treated sites than in the non-LLLT sites (2.78 [2.05-4.50] vs. 4.68 [2.41-6.86]; p = 0.035). This difference was more pronounced on day 7 (0.35 [0.24-0.56] vs. 0.72 [0.41-1.28]; p < 0.001). By day 15, complete epithelialization was observed in both the groups. The wound healing scores were comparable between the groups at all time points (p > 0.05). VAS pain scores were significantly lower in LLLT-treated sites on day 3 (p = 0.001) and day 7 (p < 0.001), with no difference on day 15 (p = 0.083).

Conclusion

Adjunctive LLLT promoted faster early epithelialization and reduced postoperative pain following gingival depigmentation. Although overall wound healing outcomes were similar between groups, LLLT appears to be a useful adjunct for improving patient comfort and early healing.

## Introduction

Gingival hyperpigmentation is a common esthetic concern, characterized by excessive melanin deposition within the basal and suprabasal layers of the gingival epithelium. Although physiological in nature and not associated with pathology, increased gingival pigmentation can compromise smile esthetics, particularly in individuals with a high smile line or fair complexion [1]. This often leads patients to seek cosmetic periodontal procedures aimed at improving gingival appearance and overall facial harmony.

Several techniques have been proposed for the management of gingival hyperpigmentation, including chemical cauterization, gingivectomy, burr abrasion, cryosurgery, laser ablation, and free gingival grafting [2,3]. Among these, scalpel-based surgical depigmentation remains a widely accepted and cost-effective technique, owing to its simplicity and minimal equipment requirements. However, this method results in a denuded connective tissue surface that heals by secondary intention and is often associated with postoperative pain, discomfort, and delayed epithelialization [3,4].

Wound healing following periodontal surgery is a complex biological process that involves hemostasis, inflammation, proliferation, and remodeling. Delayed epithelialization and postoperative discomfort remain key concerns that influence patient satisfaction and clinical outcome. In recent years, low-level laser therapy (LLLT), also referred to as photobiomodulation, has gained attention as a non-invasive adjunct for enhancing tissue repair and reducing postoperative pain [3-5]. LLLT stimulates mitochondrial activity, increases adenosine triphosphate (ATP) production, promotes fibroblast proliferation, and modulates inflammatory mediators, thereby accelerating wound healing and reducing nociception [6].

Although several studies have explored the role of LLLT in periodontal procedures, such as gingivectomy, flap surgery, and grafting procedures, evidence regarding its effectiveness following surgical gingival depigmentation remains limited and inconsistent [6]. Furthermore, most existing studies have been conducted under controlled experimental conditions, which may not accurately reflect routine clinical practice. Observational evidence is needed to evaluate the effectiveness of LLLT in real-world settings, where treatment decisions are guided by clinical judgment and patient preference.

Therefore, the present prospective observational study aimed to evaluate the clinical outcomes of adjunctive LLLT following surgical gingival depigmentation. This study aimed to assess the effect of LLLT on postoperative wound healing and pain perception. The objective of this study was to compare the degree of epithelialization, evaluate wound healing using a standardized scoring, and assess pain levels at different postoperative time intervals.

## Materials and methods

This prospective observational study was conducted at Government Dental College and Hospital, Kadapa, India, over a period of six months from January 2025 to June 2025. The study protocol adhered to the ethical principles of the Declaration of Helsinki and was approved by the Institutional Ethics Committee (Pr:002/IEC/GDCH/2024-25/01). Written informed consent was obtained from all participants prior to enrollment.

The present study was designed as a split-mouth investigation, wherein all participants underwent standard surgical gingival depigmentation as part of routine clinical care. The use of adjunctive LLLT was determined based on operator discretion and patient acceptance, representing a non-randomized allocation based on clinical decision-making rather than experimental assignment. The investigators did not intervene in assigning treatment modalities; instead, they prospectively observed and recorded clinical outcomes, including wound healing and pain perception, at sites receiving LLLT and those not receiving LLLT. This design allowed for the evaluation of outcomes under real-world clinical conditions by comparing naturally occurring exposure groups over a defined follow-up period.

The sample size for this split-mouth study was calculated a priori using the G*Power software (version 3.1.9.7; Franz Faul, Universität Kiel, Germany). Given the paired within-subject design, the Wilcoxon Signed-Rank test for matched pairs was selected as the reference statistical test for sample size computation. Using a moderate effect size (0.60) from a previous study, a two-tailed α error of 5% (α = 0.05), and a desired statistical power of 80% (1 − β = 0.80), G*Power calculated the minimum required sample size of 24 patients [7]. Accordingly, 24 patients were observed, each contributing to one non-LLLT site and one LLLT site, yielding a total of 48 study sites.

**Figure 1:**
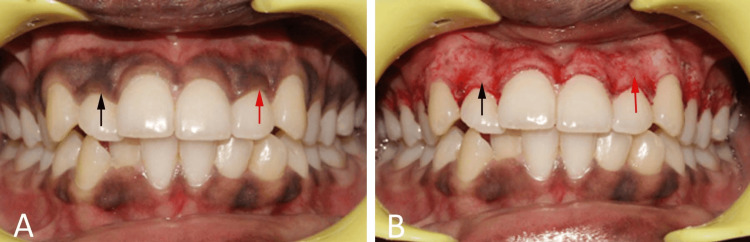
(A) Preoperative view showing bilateral gingival hyperpigmentation (denoted by a black arrow as the LLLT site and a red arrow as the non-LLLT site). (B) Intraoperative view following surgical depigmentation using the scalpel technique on the LLLT site and the non-LLLT site. LLLT, low-level laser therapy Original images of the patient, used with the patient's permission.

A total of 24 systemically healthy participants presenting with bilateral gingival hyperpigmentation in the maxillary anterior region were included in this study (Figure 1A). Participants aged between 18 and 40 years with physiological gingival melanin pigmentation, assessed using the oral pigmentation index (an open-access instrument licensed under CC BY-NC-SA 4.0) [8], with scores of 2 or 3 indicating moderate-to-severe pigmentation, and a thick gingival phenotype were included. Patients with systemic illnesses, a history of smoking or tobacco use, a history of keloid formation, pregnancy, or those outside the specified age range were excluded from the study.

All participants underwent full-mouth oral prophylaxis, including scaling and root planing, before the surgical procedure. Gingival depigmentation was performed using the surgical stripping technique under local anesthesia with 2% lignocaine hydrochloride and 1:80,000 adrenaline (Lignox®, Indoco Remedies Ltd., Mumbai, Maharashtra, India). A sterile 15C surgical blade (Swann-Morton Ltd., Sheffield, United Kingdom) was used to excise the pigmented gingival epithelium, and hemostasis was achieved following the procedure (Figure 1B).

**Figure 2:**
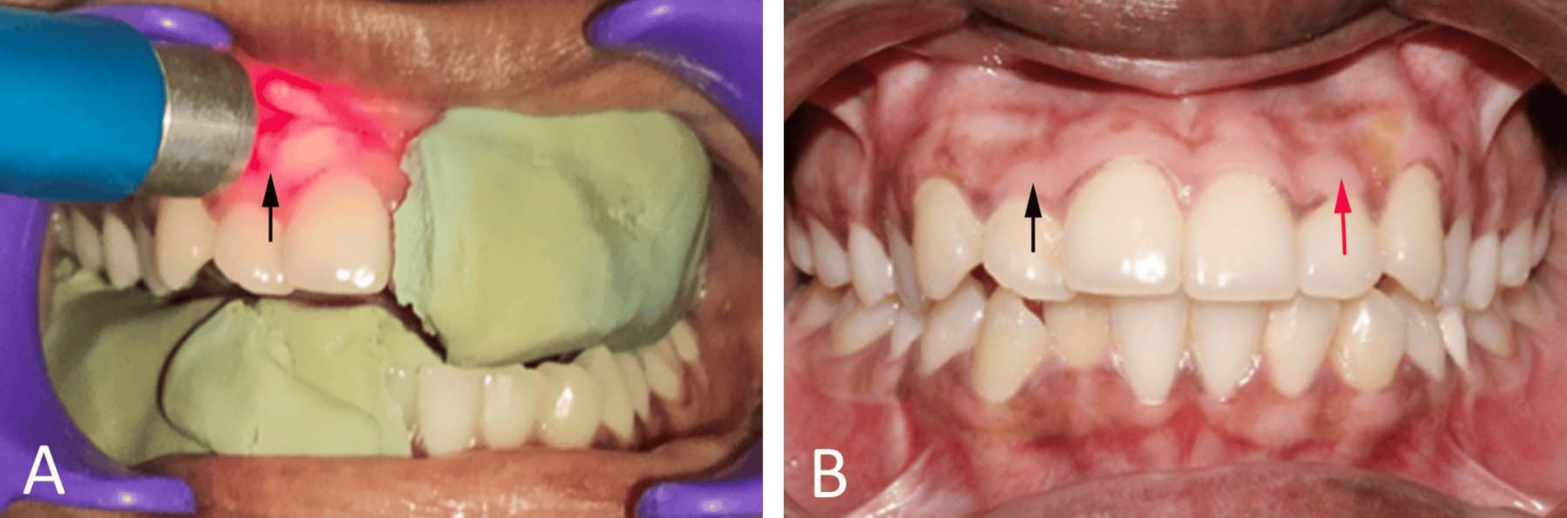
(A) Application of adjunctive LLLT at the surgical site (denoted by a black arrow as the LLLT site). (B) Postoperative healing assessment at follow-up visits (denoted by a black arrow as the LLLT site and a red arrow as the non-LLLT site). LLLT, low-level laser therapy Original images of the patient, used with the patient's permission.

Adjunctive LLLT was performed using an 810-nm diode laser operating in continuous-wave mode with an output power of 1 W. The laser was applied in a defocused, non-contact mode at an approximate distance of 1 mm from the tissue surface for 5 minutes (300 seconds) immediately after surgery and repeated once daily for seven consecutive days. The total energy delivered per treatment session was 300 J (1 W × 300 s) (Figure 2A). The contralateral sites that did not receive LLLT (non-LLLT) were used as comparison sites. To maintain the integrity of the split-mouth design and prevent unintended laser irradiation of adjacent tissues, a biocompatible silicone barrier was positioned at the midline to isolate the LLLT and non-LLLT sites during laser application.

Postoperative evaluations were performed on the 3rd, 7th, and 15th postoperative days (Figure 2B). Pain perception was assessed using a visual analog scale (VAS), an open-access patient-reported outcome measure licensed under CC BY-NC-SA 4.0 [9], with scores ranging from 0 (no pain) to 10 (worst imaginable pain). Wound healing was clinically evaluated on a five-point scale, where 1 indicated very poor healing and 5 indicated excellent healing. Wound healing score calculated based on the parameters including tissue color, bleeding, granulation tissue, epithelialization, and suppuration. Prior to its application, the scoring was reviewed by a panel of three experts in oral and maxillofacial surgery and periodontology to establish content validity. A pilot assessment was conducted on 10 patients, and intra-examiner reliability was evaluated using Cohen's kappa coefficient, demonstrating good agreement (κ = 0.82).

**Figure 3:**
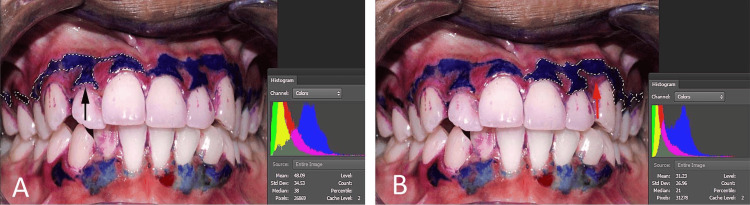
Measuring the stained area with Adobe Photoshop CS6: (A) LLLT side (denoted by a black arrow) and (B) non-LLLT side (denoted by a red arrow). LLLT, low-level laser therapy

For objective assessment of epithelialization, the treated areas were disclosed using a two-tone plaque disclosing solution, AlphaPlac® (Bombay Burmah Trading Corporation Ltd., Mumbai, Maharashtra, India), applied for 30 s, and rinsed according to the manufacturer’s instructions. Standardized intraoral photographs were obtained using a digital single-lens reflex camera (Canon DS126311; Canon Inc., Tokyo, Japan) at a fixed distance of 30 cm with standardized settings (shutter speed, 1/100; aperture, f/14; ISO, 4000; and focal length, 55 mm). The images were analyzed using Adobe Photoshop CS6 (Adobe Systems Incorporated, San Jose, CA, USA). The stained surface areas were measured in pixels using software selection tools and converted into square millimeters using reference anatomical landmarks. Calibration was performed prior to analysis using objects of known dimensions to ensure measurement accuracy (Figure 3).

Statistical analysis was performed using IBM SPSS Statistics for Windows, Version 20.0 (IBM Corp., Armonk, NY, USA). Data normality was assessed using the Shapiro-Wilk test. Descriptive statistics (mean ± standard deviation [SD] or median with interquartile range [IQR]) were computed for demographic and outcome measures. Intergroup comparisons for non-normally distributed outcomes (epithelialization, wound healing score, and VAS pain score) were conducted using the Wilcoxon signed-rank test. Intragroup comparisons across three time points (days 3, 7, and 15) were performed using the Friedman test, followed by post-hoc pairwise comparisons using the Wilcoxon signed-rank test and Bonferroni correction (adjusted α = 0.016). Statistical significance was set at p < 0.05. All tests were two-tailed.

## Results

A total of 24 participants were included in the study, each contributing two surgical sites, resulting in 48 evaluated sites. The demographic characteristics of the study population are shown in Table 1. The mean age of participants was 28.52 ± 5.60 years, with 14 males and 10 females. All participants completed the follow-up period without any reported complications.

**Table 1:** Demographic characteristics of the study population. Values are expressed as mean ± SD for continuous variables and as frequency (percentage) for categorical variables. Total number of patients (N) = 24, total number of sites (n) = 48.

Parameter	Category	Participants
Age (years), mean ± SD	Overall	28.52 ± 5.60
	Male	29.45 ± 6.20
	Female	28.12 ± 5.42
Sex, n (%)	Male	14 (58%)
	Female	10 (42%)

Normality testing using the Shapiro-Wilk test revealed that the outcome variables were not normally distributed; therefore, non-parametric statistical tests were applied. Inter-site comparisons between sites receiving adjunctive LLLT and those not receiving LLLT were performed using the Wilcoxon signed-rank test, as summarized in Table 2. For epithelialization, a statistically significant difference was observed between the two groups at early postoperative time points. On day 3, sites receiving LLLT demonstrated a significantly lower residual wound area compared to non-LLLT sites (p = 0.035). This difference was more pronounced on day 7 (p < 0.001), indicating enhanced epithelialization at the LLLT-treated sites. By day 15, complete epithelialization was achieved at all sites, with no measurable wound areas remaining in either group (Table 2).

**Table 2:** Inter-site comparison of outcome measures between LLLT and non-LLLT sites. Values are expressed as median (IQR). *A p-value of <0.05 denotes statistical significance. Inter-site comparisons were performed using the Wilcoxon signed-rank test. "—" denotes that no statistical test is needed. IQR, interquartile range; LLLT, low-level laser therapy; VAS, visual analog scale; W, Wilcoxon signed-rank test statistic

Outcome	Day	Non-LLLT, median (IQR)	LLLT, median (IQR)	Test statistics (W)	p-Value
Epithelialization (mm^2^)	3	4.68 (2.41–6.86)	2.78 (2.05–4.5)	69	0.035
	7	0.72 (0.41–1.28)	0.35 (0.24–0.56)	0	<0.001*
	15	0 (0–0)	0 (0–0)	—	—
Wound healing score	3	2 (2–2)	2 (2–3)	7	0.414
	7	4 (4–4)	4 (4–4)	0	0.157
	15	5 (5–5)	5 (5–5)	0	0.317
VAS pain score	3	5 (5–6)	4 (3–4)	27	0.001*
	7	3 (2–3)	1 (1–1.25)	18	<0.001*
	15	0 (0–0)	0 (0–0)	0	0.083

Assessment of wound healing using the customized wound-healing score showed progressive improvement in both groups over time. However, no statistically significant differences were observed between LLLT-treated and non-LLLT sites at any time point (day 3, p = 0.414; day 7, p = 0.157; and day 15, p = 0.317), indicating comparable overall healing patterns (Table 2). Pain perception, evaluated using the VAS, demonstrated significantly lower pain scores at sites receiving LLLT in the early postoperative stages. On day 3, the LLLT group showed significantly reduced pain compared with the non-LLLT group (p = 0.001). This difference remained highly significant on day 7 (p < 0.001). By day 15, pain had resolved in both groups, with no statistically significant difference observed (p = 0.083) (Table 2).

Intragroup comparisons over time, analyzed using the Friedman test, revealed statistically significant improvements in all outcome measures in both groups (Table 3). Epithelialization showed a consistent reduction in wound area from day 3 to day 15 (p < 0.001), while wound healing scores improved progressively, reflecting the transition from the early healing stages to complete tissue maturation (p < 0.001). Similarly, the VAS pain scores demonstrated a significant decline over time in both groups (p < 0.001).

**Table 3:** Intragroup comparison of outcome measures over time. Values are expressed as median (IQR). The Friedman test (χ²) was used for repeated measures within each group. *A p-value of <0.05 denotes statistical significance. IQR, interquartile range; LLLT, low-level laser therapy; VAS, visual analog scale

Outcome variable	Group	Median (IQR)	Median (IQR)	Median (IQR)	Test statistics (χ²)	p-Value
		Day 3	Day 7	Day 15		
Epithelialization (mm^2^)	Non-LLLT	4.68 (2.41–6.86)	0.72 (0.41–1.28)	0 (0–0)	48.0	<0.001*
	LLLT	2.78 (2.05–4.49)	0.35 (0.24–0.56)	0 (0–0)	47.0	<0.001*
Wound healing score	Non-LLLT	2 (2–2)	4 (4–4)	5 (5–5)	46.2	<0.001*
	LLLT	2 (2–3)	4 (4–4)	5 (5–5)	46.6	<0.001*
VAS score	Non-LLLT	5 (5–6)	3 (2–3)	0 (0–0)	48.0	<0.001*
	LLLT	4 (3–4)	1 (1–1.25)	0 (0–0)	47.5	<0.001*

Post-hoc pairwise comparisons were performed using the Wilcoxon signed-rank test with Bonferroni correction (Table 4). For both groups, epithelialization area and VAS scores showed significant reductions from day 3 to day 7, day 3 to day 15, and day 7 to day 15 (all p < 0.001). Similarly, wound healing scores demonstrated significant improvement across all pairwise time-point comparisons (all p < 0.001).

**Table 4:** Post-hoc analysis with the Wilcoxon signed-rank test. Values are presented as z-statistics from the Wilcoxon signed-rank test. *Statistical significance was determined using the Bonferroni-adjusted significance threshold of p < 0.016 to account for multiple pairwise comparisons. LLLT, low-level laser therapy; VAS, visual analog scale

Outcome variable	Group	Day 3 vs day 7		Day 3 vs day 15		Day 7 vs day 15	
		Test statistics (z)	p-Value	Test statistics (z)	p-Value	Test statistics (z)	p-Value
Epithelialization (mm^2^)	Non-LLLT	-4.20	<0.001*	-4.30	<0.001*	-3.90	<0.001*
	LLLT	-4.10	<0.001*	-4.30	<0.001*	-3.80	<0.001*
Wound healing score	Non-LLLT	-4.15	<0.001*	-4.30	<0.001*	-3.95	<0.001*
	LLLT	-4.05	<0.001*	-4.25	<0.001*	-3.90	<0.001*
VAS score	Non-LLLT	-4.10	<0.001*	-4.30	<0.001*	-4.00	<0.001*
	LLLT	-4.25	<0.001*	-4.35	<0.001*	-3.95	<0.001*

**Figure 4:**
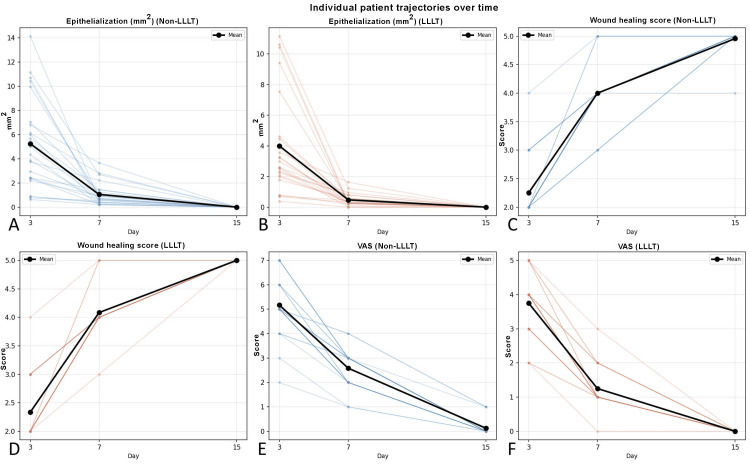
Changes in outcome measures over time. Spaghetti plots illustrating individual patient trajectories for epithelialization (A, B), wound healing score (C, D), and VAS pain scores (E, F) across postoperative days 3, 7, and 15 for non-LLLT and LLLT sites. Each line represents an individual site, demonstrating temporal trends in healing and pain reduction. LLLT, low-level laser therapy; VAS, visual analog scale

The temporal trends and individual variations in epithelialization, wound healing score, and pain scores are illustrated using spaghetti plots in Figure 4. These plots demonstrate a consistent pattern of improvement across all participants, with a more rapid decline in wound area and pain scores observed at sites receiving LLLT, particularly during the early postoperative period. Overall, the findings suggest that adjunctive LLLT enhances early epithelialization and significantly reduces postoperative pain, whereas overall wound healing progression remains comparable between sites.

## Discussion

This prospective observational study evaluated the effect of adjunctive LLLT on wound healing and postoperative pain following surgical gingival depigmentation. The findings demonstrated that LLLT was associated with significantly enhanced early epithelialization and reduced postoperative pain, whereas overall wound healing progression, as assessed by a clinical index, remained comparable between sites.

A key finding of this study was the significantly improved epithelialization observed in LLLT-treated sites during the early postoperative phase (days 3 and 7). This can be attributed to the biostimulatory effects of photobiomodulation on cellular activities. LLLT enhances mitochondrial function, leading to increased adenosine triphosphate (ATP) production, which, in turn, promotes fibroblast proliferation, collagen synthesis, and epithelial cell migration. These cellular events accelerate wound closure and reduce the residual wound area [6,10]. Similar findings were reported by Ozcelik et al. [11], who demonstrated enhanced epithelial healing following gingivectomy with adjunctive LLLT. Likewise, Kohale et al. [7] observed significantly faster wound healing and improved patient outcomes at laser-treated sites, supporting the present findings.

Despite the observed improvement in epithelialization, no statistically significant differences were found between the groups in terms of wound healing score. This may be explained by the inherent limitations of categorical clinical indices that assess healing in broader stages rather than capturing subtle quantitative differences. Although LLLT may accelerate early cellular events and epithelial migration, both groups ultimately follow similar biological healing pathways, resulting in comparable clinical healing scores over time. This observation is consistent with previous studies that reported improved early healing parameters without significant differences in the overall clinical healing indices [12,13].

Pain reduction was another significant outcome in the present study, with LLLT-treated sites demonstrating lower VAS scores on days 3 and 7. The analgesic effect of LLLT is well documented and is primarily mediated through modulation of nerve conduction and inflammatory pathways. LLLT reduces the release of pro-inflammatory mediators, such as prostaglandins and cytokines, while enhancing endorphin release, thereby decreasing nociceptor sensitivity. Additionally, it may inhibit transmission to the A-delta and C nerve fibers, leading to reduced pain perception [14]. These findings are in agreement with those reported in previous studies, which observed significant reductions in postoperative pain following depigmentation procedures with LLLT [4,15]. Similarly, previous reviews have highlighted the role of photobiomodulation in minimizing postoperative discomfort in various periodontal procedures [6,16].

Intragroup analysis further confirmed that all clinical parameters improved significantly over time in both the groups, reflecting the natural progression of wound healing from inflammation to proliferation and maturation. Complete epithelialization and resolution of pain by day 15 in all participants indicated that surgical depigmentation is a predictable and effective procedure, with LLLT serving as a beneficial adjunct to enhance early healing dynamics and patient comfort.

Clinical implications

Adjunctive LLLT may enhance early epithelialization and significantly reduce postoperative pain following gingival depigmentation. Its non-invasive nature and ease of application make it a useful supportive adjunct in routine periodontal practice, particularly for improving patient comfort and early healing outcomes.

Limitations

The observational design of the study without randomization may introduce selection bias and limit causal inferences. The small sample size and short follow-up period restrict the generalizability and long-term evaluation. Additionally, the use of a customized wound-healing score and subjective pain assessment may affect comparability and introduce measurement variability. An additional limitation of this study is that detailed laser dosimetry parameters, including spot size, beam area, irradiance, and energy density (fluence), were not documented at the time of treatment. Although the wavelength, output power, irradiation time, and treatment frequency were standardized for all participants, the absence of these parameters may limit the reproducibility and direct comparison of the findings with other photobiomodulation studies. Future studies should report laser parameters in accordance with the current photobiomodulation reporting recommendations.

## Conclusions

Within the limitations of this prospective observational study, adjunctive LLLT demonstrated a beneficial effect in enhancing early epithelialization and significantly reducing postoperative pain after surgical gingival depigmentation. However, the overall wound healing progression, as assessed by the clinical healing score, was comparable between the sites. These findings suggest that LLLT serves as an effective supportive adjunct for improving early healing dynamics and patient comfort, without altering the final healing outcome.
